# 6-mercaptopurine promotes energetic failure in proliferating T cells

**DOI:** 10.18632/oncotarget.17889

**Published:** 2017-05-16

**Authors:** Ana A. Fernández-Ramos, Catherine Marchetti-Laurent, Virginie Poindessous, Samantha Antonio, Pierre Laurent-Puig, Sylvie Bortoli, Marie-Anne Loriot, Nicolas Pallet

**Affiliations:** ^1^ INSERM UMR-S 1147, Centre Universitaire des Saints-Pères, 75006 Paris, France; ^2^ Université Paris Descartes, Sorbonne Paris Cité, 75006 Paris, France; ^3^ INSERM UMR-S 1124, Centre Universitaire des Saints-Pères, 75006 Paris, France; ^4^ Assistance Publique-Hôpitaux de Paris, Hôpital Européen Georges Pompidou, Service de Biochimie, 75015 Paris, France

**Keywords:** 6-mercaptopurine, energetic failure, metabolic checkpoints, acute lymphoblastic leukemia, Jurkat T cell line

## Abstract

The anticancer drug 6-mercaptopurine (6-MP) inhibits *de novo* purine synthesis and acts as an antiproliferative agent by interfering with protein, DNA and RNA synthesis and promoting apoptosis. Metabolic reprogramming is crucial for tumor progression to foster cancer cells growth and proliferation, and is regulated by mechanistic target of rapamycin (mTOR) and AMP-activated protein kinase (AMPK) as well as the oncogenes Myc and hypoxia inducible factor 1α (HIF-1α). We hypothesized that 6-MP impacts metabolic remodeling through its action on nucleotide synthesis. The aim of our study is to provide a comprehensive characterization of the metabolic changes induced by 6-MP in leukemic T cells. Our results indicate that exposition to 6-MP rapidly reduces intracellular ATP concentration, leading to the activation of AMPK. In turn, mTOR, an AMPK target, was inhibited, and the expression of HIF-1α and Myc was reduced upon 6-MP incubation. As a consequence of these inhibitions, glucose and glutamine fluxes were strongly decreased. Notably, no difference was observed on glucose uptake upon exposition to 6-MP. In conclusion, our findings provide new insights into how 6-MP profoundly impacts cellular energetic metabolism by reducing ATP production and decreasing glycolytic and glutaminolytic fluxes, and how 6-MP modifies human leukemic T cells metabolism with potential antiproliferative effects.

## INTRODUCTION

The thiopurine drugs, including azathioprine, 6-mercaptopurine (6-MP) and 6-thioguanine (6-TG), are purinergic antimetabolite agents with profound antiproliferative effects [1–6]. Azathioprine is converted into 6-MP, whose enzymatically driven metabolites 6-thioguanine nucleotides and 6-methylmercaptopurine interfere with RNA, DNA and protein synthesis as well as promote apoptosis of proliferating T cells [1–3, 6–9]. Although the precise molecular mechanism involved in the inhibition of T cells still remain unclear, thiopurines are prescribed for the treatment of numerous diseases involving T cells activation and proliferation [1–6]. Azathioprine was the first immunosuppressive agent approved to prevent rejection of solid organ transplants and is an effective therapy to maintain remission of autoimmune diseases [2, 3, 5, 10]. Specifically, 6-MP is widely prescribed to block tumor growth of childhood acute lymphocytic leukemia (ALL) [1, 4, 5, 7, 8, 10–15].

Numerous mechanisms are involved in the antiproliferative effects of 6-MP, which include its incorporation into nucleic acids chains (leading to DNA damage and apoptosis) and its ability to inhibit *de novo* purine synthesis [1–6, 10]—which is crucial for lymphocyte proliferation because these cells rely more on *de novo* pathway than on the salvage pathway [5, 16, 17]. 6-MP can also inhibit *de novo* biosynthesis of ATP and GTP [18]. In addition, recent evidence indicates that 6-MP inhibits the phosphatidylinositol 3 kinase (PI3K) / mammalian target of rapamycin (mTOR) signaling pathway [8], suggesting that these drugs might interfere with metabolic checkpoints and impact metabolic reprogramming in normal T cells and cancer [19]. In line with its possible role in cell metabolic reprogramming, 6-MP regulates the activity of members of the orphan nuclear receptor NR4A family, which acts as key transcriptional regulators of glucose and lipid metabolism [20]. In addition, 6-MP modifies the transcriptional activity of hypoxia inducible factor 1α (HIF-1α) [21] and inhibits enzymes implicated in glycolysis, such as phosphofructokinase 2 (PFK2) and hexokinase (HK) [22].

Metabolic reprogramming promotes glycolysis, glutaminolysis, and biosynthesis of nucleotides and lipids to support cell growth and proliferation [19, 23], and, as such, is a major feature of cancer cells. To produce ATP, proliferating cells shift glucose metabolism from oxidative phosphorylation (OXPHOS) to aerobic glycolysis [24–27], a process far less efficient than OXPHOS [19, 23–25, 27, 28] but one that generates biosynthetic precursors through the pentose phosphate pathway (PPP) [19, 23, 26] and facilitates the production of a pool of purine and pyrimidine nucleotides to support cancer cell growth and proliferation [19, 23]. Critical regulators of metabolic reprogramming in proliferating cells, referred to as metabolic checkpoints, include mTOR, AMP-activated protein kinase (AMPK) and the oncogenes Myc and HIF-1α [19, 23].

We conducted the present study to test whether 6-MP impacts T cell metabolism. We performed a comprehensive analysis of the metabolic changes promoted by 6-MP in proliferating T cells, and we demonstrated that 6-MP inhibits ATP synthesis and promotes global shutdown of glucose metabolism, leading to an energetic distress.

## RESULTS

### 6-mercaptopurine promotes T cell cycle arrest and apoptosis

**Figure 1:**
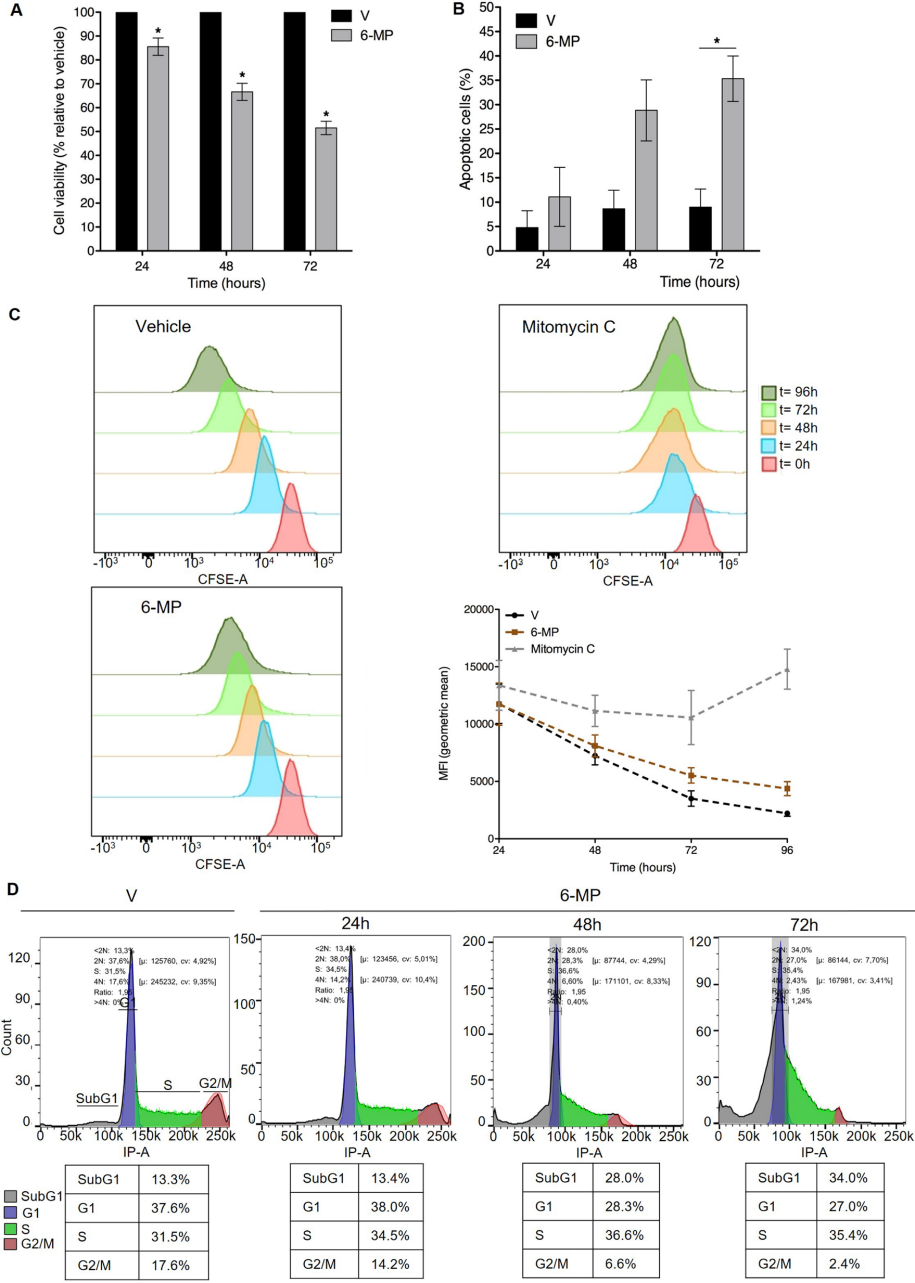
6-mercaptopurine promotes T cell cycle arrest and apoptosis (**A**) Cell viability upon exposition to 50 μM 6-MP in Jurkat cells after 24 h, 48 h and 72 h versus vehicle (V). The data are from four independent experiments. (**B**) The percentage of apoptotic cells after 24 h, 48 h or 72 h with vehicle (V) or 50 μM 6-MP. The data are from three independent experiments. (**C**) Cell proliferation of CFSE-stained Jurkat cells. Cells were incubated for 24, 48, 72 or 96 h with 50 μM 6-MP, vehicle (V) or mitomycin C (positive control). The data are from three independent experiments. (**D**) Cell cycle analysis of PI-stained Jurkat cells. Cells were incubated for 24, 48, or 72 h with 50 μM 6-MP or vehicle (V). The data are from three independent experiments.

Using a standard concentration of 50 μM [8, 20, 21], we first determined the impact of 6-MP on Jurkat T cells proliferation and viability. 6-MP significantly reduced cell viability in a time-dependent manner (Figure 1A), and after a 48-h incubation, 50 μM 6-MP reduced viability by approximately 30% compared with cells treated with vehicle (V). Since a diminution in cell viability can result from reduced proliferation and/or increased cell death, we assessed the impact of 6-MP on apoptosis. We observed a time-dependent increase in apoptosis, and after a 48-h incubation with 50 μM 6-MP, 30% of the cells were apoptotic (Figure 1B), which confirms that 6-MP promotes apoptosis [29–31]. Furthermore, 6-MP induces autophagosomes accumulation after 48 h and 72 h of incubation compared to vehicle (Supplementary Figure 1). Indeed, LC3 immunoblotting indicates that upon 6-MP incubation, the cytosolic form of LC3 (LC3-I) is converted into the LC3-phosphatidylethanolamine conjugate (LC3-II), which is recruited to autophagosomal membranes. In addition to promoting cell death, 6-MP slightly reduced cell proliferation in a time-dependent manner based on the measurement of the Carboxyfluorescein succinimidyl ester (CFSE) dilution (Figure 1C) using mitomycin C as a positive control. In line with a reduction in proliferation, 6-MP promoted an accumulation of cells stalled in sub-G1 phase in a time-dependent manner, with 34% of cells in the sub-G1 phase (fragmented nuclei) after 72 h compared with 13% in the vehicle-incubated cells (Figure 1D), whereas the proportion of cells in the G1 phase decreased from 38% to 27%, as expected. This suggests that 6-MP induces cell cycle alterations by blocking cell cycle at sub-G1 phase. Together, these results indicate that 6-MP blocks cell cycle progression and promotes T cell apoptosis.

### 6-mercaptopurine induces ATP, ADP and AMP depletion and promotes energetic stress

**Figure 2:**
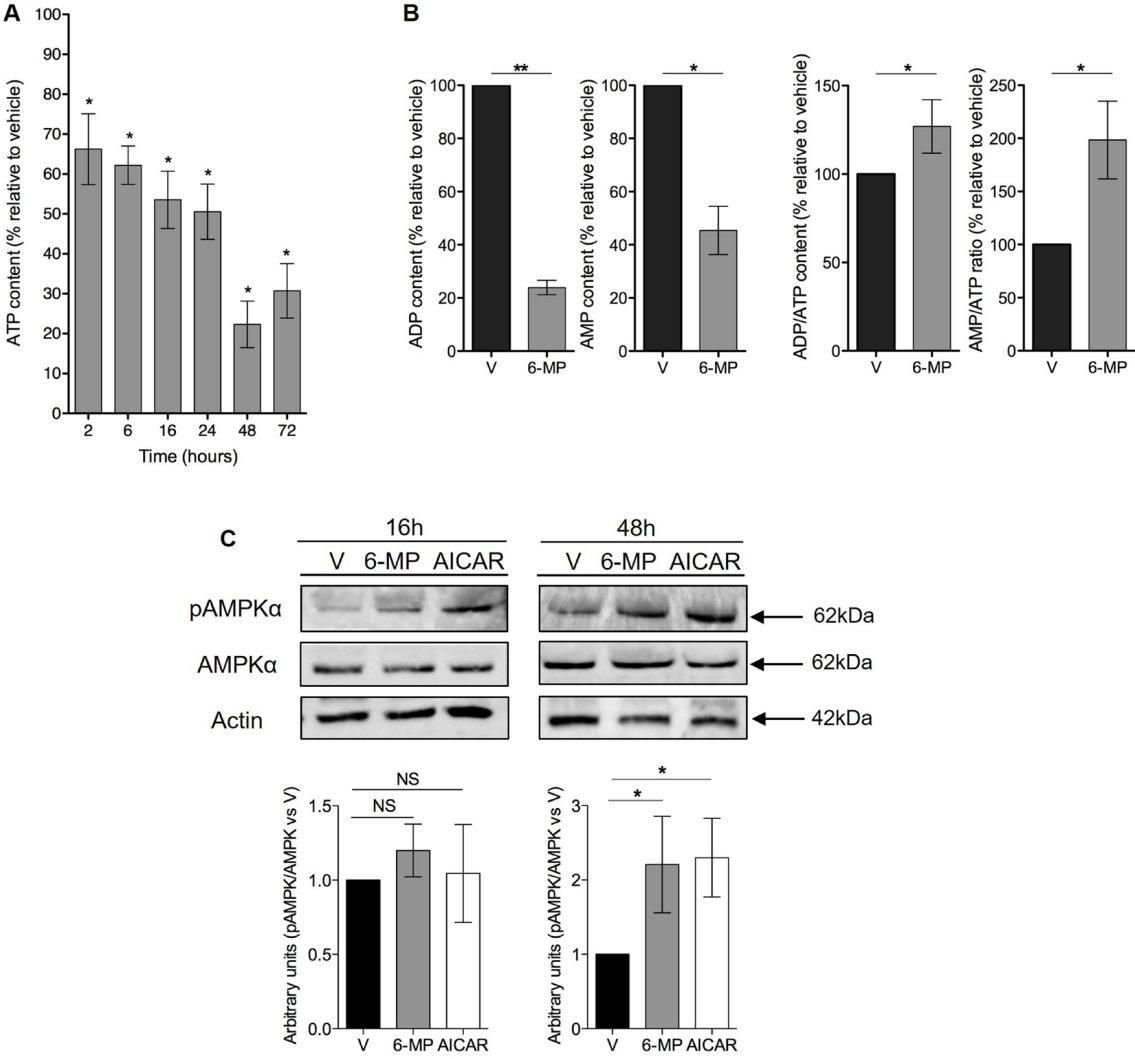
6-mercaptopurine induces ATP depletion and promotes an energetic stress (**A**) The percentage of ATP content relative to vehicle (V) after 2, 6, 16, 24, 48 or 72 h of incubation with 50 μM 6-MP. The data are from four independent experiments. (**B**) The percentage of ADP and AMP content and ADP/ATP and AMP/ATP ratio relative to vehicle (V) after 48 h of exposition to 50 μM 6-MP. The data are from four independent experiments (**C**) (Top) Immunoblot of phospho-AMPK, total AMPK and actin protein expression in a human T lymphocyte leukemia cell line (Jurkat) after 16-h or 48-h incubation with 50 μM 6-MP or vehicle (V). AICAR was used as a positive control. The immunoblot is representative of four independent experiments. (Bottom) Histograms representing the densitometric analysis of the immunoblots.

**Figure 3:**
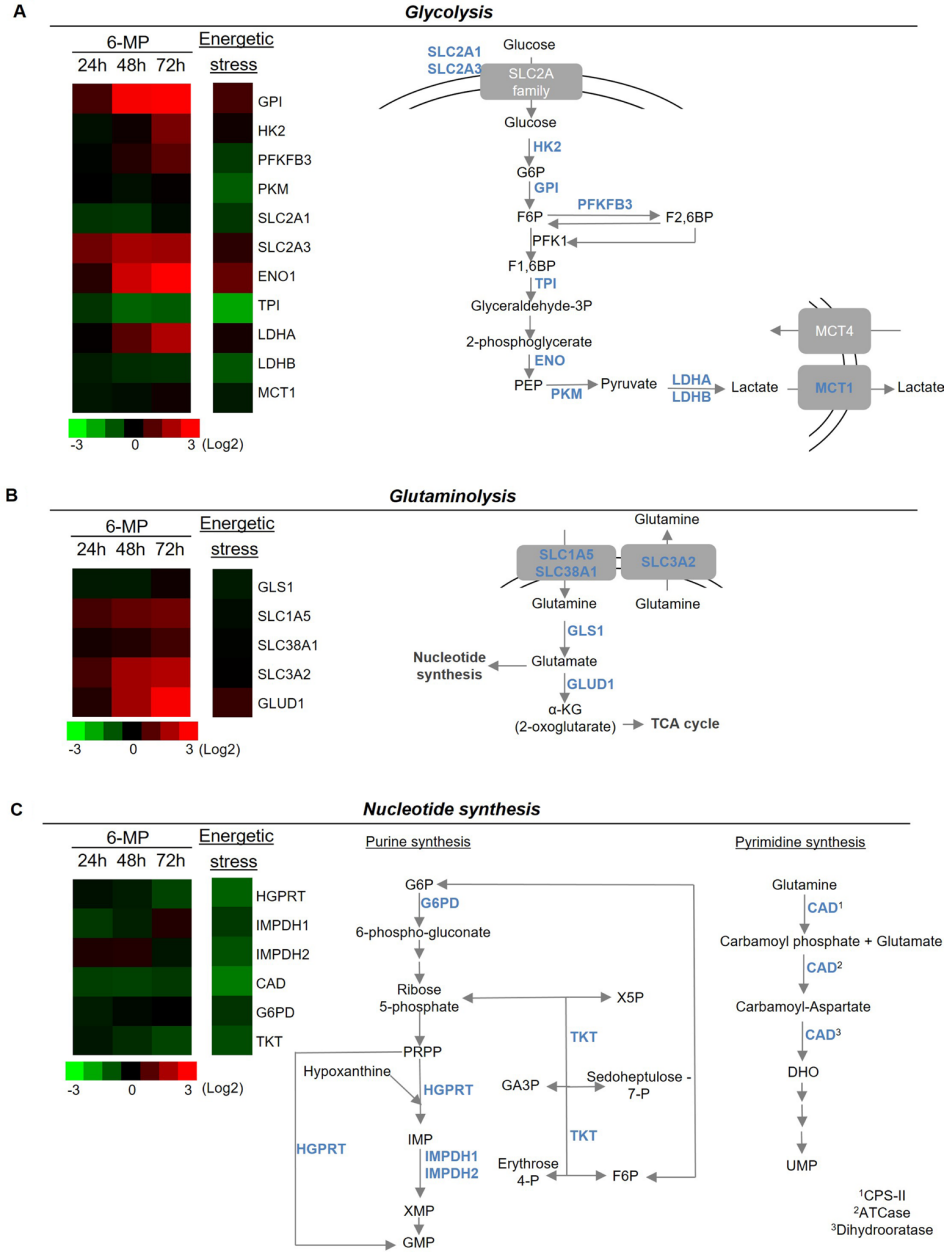
6-mercaptopurine modifies the expression of genes implicated in glycolysis, glutaminolysis and nucleotide synthesis (Left) Heat map representation of a transcriptomic profile of genes implicated in glycolysis (**A**), glutaminolysis (**B**) and nucleotide synthesis (**C**) after either 24, 48 or 72 h of incubation with 50 μM 6-MP or 16 h of energetic stress (nutrient and serum-free medium). A schematic representation of genes implicated in glycolysis, glutaminolysis and nucleotide synthesis is shown at the right. The data are from four independent experiments. ATCase: aspartate carbamoyltransferase; CAD: carbamoyl-phosphate synthetase 2, aspartate transcarbamylase, and dihydroorotase; CPS-II: carbamoyl phosphate synthetase II; DHO: dihydroorotate; ENO1: enolase 1; F1,6BP: fructose1,6-biphosphate; F6P: fructose-6-phosphate; G6P: glucose-6-phosphate; G6PD: glucose-6-phosphate dehydrogenase: GA3P: glyceraldehyde 3-phosphate; GLS1: glutaminase 1; GLUD1: glutamate dehydrogenase 1; GMP: guanosine monophosphate; GPI: glucose-6-phosphate isomerase; HGPRT: hypoxanthine-guanine phosphoribosyltransferase; HK2: hexokinase II; IMP: inosine monophosphate; IMPDH1: inosine 5′-monophosphate dehydrogenase 1; IMPDH2: inosine 5′-monophosphate dehydrogenase 2; LDHA: lactate dehydrogenase A; LDHB: lactate dehydrogenase B; MCT1: monocarboxylate transporter 1; MCT4: monocarboxylate transporter 4; PEP: phosphoenolpyruvate; PKFKB3: 6-phosphofructo-2-kinase/fructose-2,6-biphosphatase 3; PKM: pyruvate kinase muscle; PRPP: phosphoribosyl pyrophosphate; SLC1A5: solute carrier family 1, member 5; SLC2A1: solute carrier family 2, member 1; SLC2A3: solute carrier family 2, member 3; SLC38A1: solute carrier family 38, member 1; SLC3A2: solute carrier family 3, member 2; TCA cycle: tricarboxylic acid; TKT: transketolase; TPI: triosephosphate isomerase; UMP: uridine monophosphate; X5P: xylulose-5-phosphate.

Since 6-MP interferes with purine metabolism, we hypothesized that 6-MP reduces ATP production thereby promoting energetic stress and impacting T cell metabolism. 6-MP induced a significant reduction in intracellular ATP content as early as 2 h after initiating exposition, and this reduction became progressively more pronounced through 48 h (Figure 2A). Consistent with an impact of 6-MP on nucleotide synthesis, ADP and AMP levels are also significantly reduced at 48-h exposition (Figure 2B). Moreover, ADP/ATP ratio and AMP/ATP ratio are significantly increased after 48 h of incubation with 6-MP. Because AMPK is a cellular energy sensor that depletion or with high AMP/ATP ratio, we examined the phosphorylation status of the threonine 172 located in the catalytic subunit of AMPK by immunoblotting (Figure 2C) [32–34]. We analyzed the expression levels of phospho-AMPK and total AMPK after 16- and 48-h incubations with either 6-MP or vehicle (V). 5-Aminoimidazole-4-carboxamide ribonucleotide (AICAR), an analogue of AMP, activates AMPK and was used as positive control. After 16-h exposition to 6-MP, no statistical difference was observed in the activation of AMPK compared to vehicle (V). However, after 48 h of incubation with 6-MP, phosphorylation of AMPK was significantly increased. Having shown that 6-MP promotes ATP depletion and the consequent activation of AMPK, we reasoned that metabolic modifications likely occur in response to this energetic stress. 6-MP significantly altered the level expression of genes involved in glycolysis and glutaminolysis (Figure 3A, 3B and Supplementary Figures 2 and 3). Glycolysis-related genes such as glucose-6-phosphate isomerase (GPI); hexokinase 2 (HK2); 6-phosphofructo-2-kinase/fructose-2,6-biphosphatase (PFKFB3); solute carrier family 2, member 3, Glut3 (SLC2A3); enolase 1 (ENO1) and lactate dehydrogenase (LDHA) as well as glutaminolysis-related genes including solute carrier family 1, member 5 (SLC1A5); solute carrier family 38, member 1 (SLC38A1); solute carrier family 3, member 2 (SLC3A2) and glutamate dehydrogenase 1 (GLUD1) were significantly upregulated. On the other hand, the expression of genes involved in purine or pyrimidine synthesis (Figure 3C) including hypoxanthine-guanine phosphoribosyltransferase (HGPRT), carbamoyl-phosphate synthetase 2, aspartate transcarbamylase, and dihydrooratase (CAD) and transketolase (TKT) were significantly reduced.

Interestingly, this profile is similar (albeit with higher intensity) to that of induced by energetic stress (e.g., glucose/glutamine starvation), during which GPI, SLC2A3, ENO1, LDHA and of GLUD1 are upregulated, and genes involved in the PPP pathway and the synthesis of purines (HGPRT, and inosine 5′-monophosphate dehydrogenase (IMPDH) 1 and 2, and TKT) and in the synthesis of pyrimidines (CAD) are significantly downregulated, which reinforces the assumption that 6-MP promotes energetic stress in proliferating T cells. Even the physiological impact of these transcriptomic changes still requires further elucidation, it is conceivable that these expression profiles correspond to metabolic rewiring that would optimize glycolytic and glutaminolytic pathways (e.g., overexpression of glucose and glutamine transporters and key enzymes of glycolysis) to produce ATP in response to an energetic stress. Together, our findings support that, by inhibiting ATP synthesis, 6-MP promotes energetic stress resulting into the altered expression of genes involved in nucleotide synthesis as well as glucose and glutamine metabolism.

### 6-mercaptopurine inhibits metabolic checkpoints and decreases glycolytic and glutaminolytic fluxes

**Figure 4:**
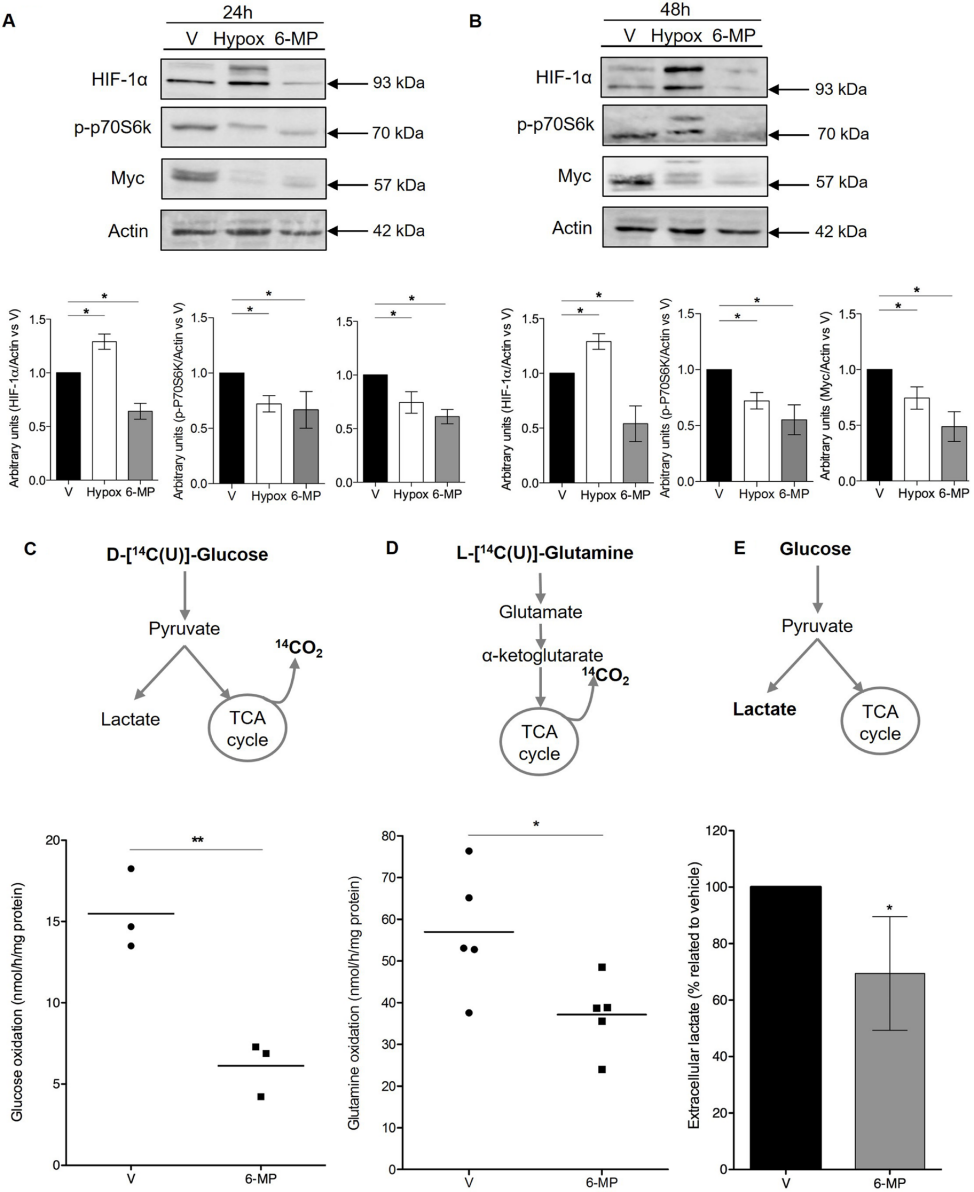
6-mercaptopurine inhibits metabolic checkpoints and decreases glycolytic and glutaminolytic flux (**A** and **B**, Top) Immunoblot representing phospho-p70S6K (70 kDa ribosomal protein S6 kinase 1), HIF-1α (hypoxia inducible factor 1α), Myc, and actin protein expression in a human T lymphocyte leukemia cell line (Jurkat) after 24 h (A) or 48 h (B) of incubation with 50 μM 6-MP or vehicle (V). A 24-h exposure to hypoxia was used as a control for HIF-1α. The immunoblot is representative of four independent experiments. (Bottom) Histograms representing the densitometric analysis of the immunoblots. (**C**) Glycolytic and (**D**) glutaminolytic flux after a 48-h incubation with 50 μM 6-MP or vehicle (V). (Top) Schematic representation of the procedure. (Bottom) Box-and-whisker plots representing the glucose rate (C) and glutamine rate (D) by nmol of glucose or glutamine metabolized per h per mg of protein, respectively, after a 48-h incubation with 50 μM 6-MP or vehicle (V). The data are from three or five independent experiments. (**E**) Histograms representing the results of percentage of extracellular lactate as compared to vehicle. Jurkat cells were incubated with 50 μM 6-MP or vehicle (V) for 48 h, and extracellular lactate was measured using a Lactate Colorimetric/Fluorometric Assay Kit. The data are from four independent experiments.

Having shown that 6-MP promotes energetic stress and impacts the expression of genes involved in glycolysis (i.e., the degradation of glucose into pyruvate [35]) and glutaminolysis, we hypothesized that 6-MP modulates the expression of metabolic checkpoints, leading to alteration of cell metabolism. The expression levels of HIF-1α and Myc were significantly decreased in T cells incubated with 6-MP for 24 h and 48 h (Figure 4A, 4B), and in the same experimental conditions, 6-MP also significantly reduced p70S6K phosphorylation, indicating that mTOR activity was inhibited. These effects were maintained up to 72 h of exposition to 6-MP (Supplementary Figure 4). Consistent with the downregulation of metabolic checkpoints in T cells, the metabolic activity of T cells was profoundly impacted by 6-MP. As measured by the rate of CO_2_ released from U-^14^C-glucose and U-^14^C-glutamine, glucose and glutamine oxidation capacities through the TCA (tricarboxylic acid cycle) followed by OXPHOS (Figure 4C, 4D), were significantly decreased by 60% and 35%, respectively, after a 48-h incubation with 6-MP, indicating that the oxidation capacities of Jurkat cells from glucose and glutamine substrates were drastically reduced upon exposition to 6-MP. Since 6-MP inhibited glucose degradation from glycolysis to TCA and OXPHOS, glucose metabolism could be redirected toward lactic acid production (i.e., pyruvate conversion into lactate [35]). However, the production of lactate, which is a marker of aerobic glycolysis (Warburg effect [25, 28]) and is reflected by the extracellular lactate concentration, was significantly decreased by 30% (Figure 4E), indicating that aerobic glycolysis is also inhibited by 6-MP. Together, these data indicate that 6-MP inhibits the metabolic checkpoints mTOR, HIF-1α and Myc and decreases both the glucose and glutamine catabolism through glycolysis and the TCA cycle.

### 6-mercaptopurine does not modify either glucose uptake or Glut1 and Glut3 expression

**Figure 5:**
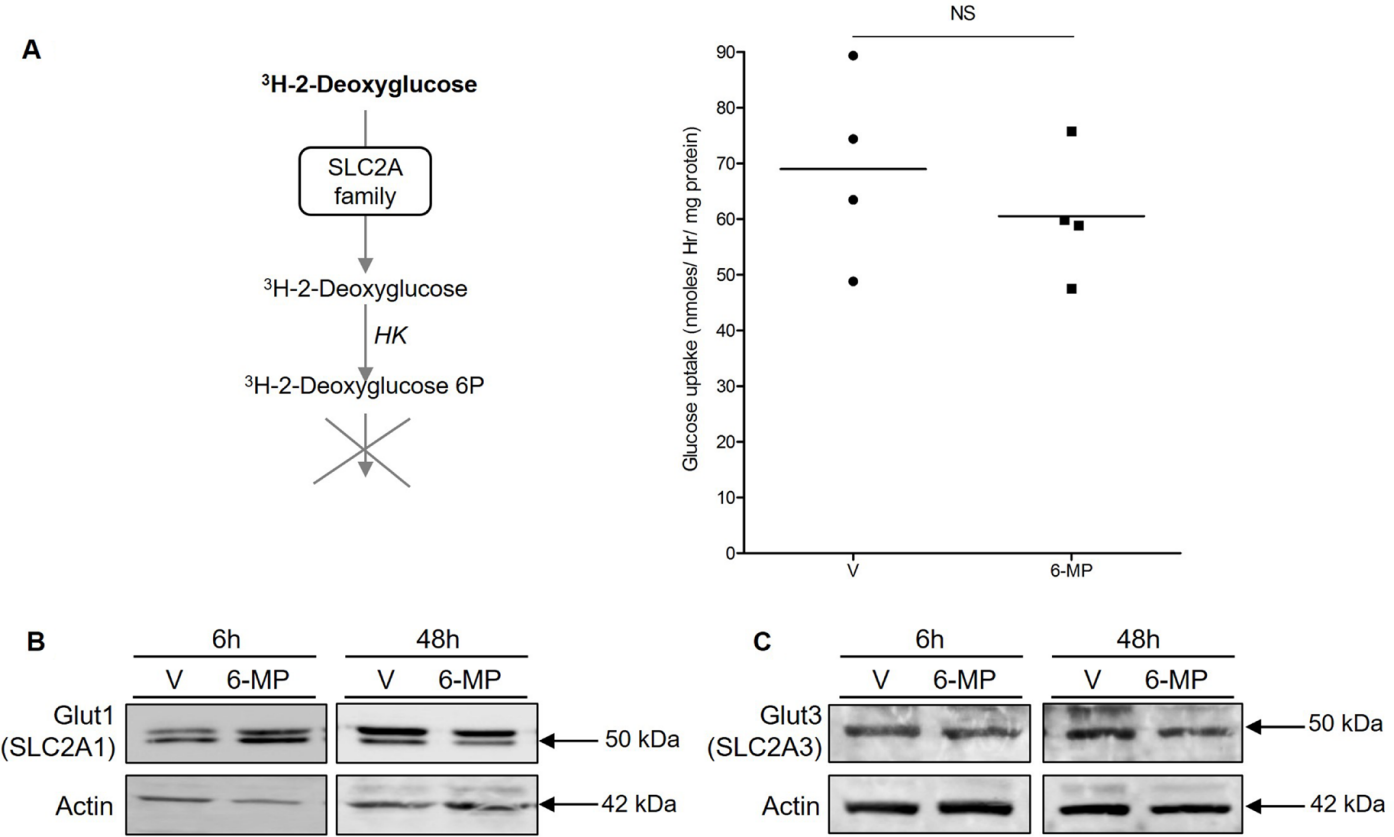
6-mercaptopurine does not modify either glucose uptake or Glut1 and Glut3 expression (**A**) (Left) Schematic representation of the procedure. (Right) Jurkat cells were exposed to 50 μM 6-MP or vehicle (V) for 48 h with, and the quantity of ^3^H-2-deoxyglucose was analyzed. The data are from four independent experiments. (**B**) Immunoblot representing Glut1 (SLC2A1) and actin protein expression in a human T lymphocyte leukemia cell line (Jurkat) after a 6-h or 48-h incubation with 50 μM 6-MP or vehicle (V). The immunoblot is representative of three independent experiments. (**C**) Immunoblot representing Glut3 (SLC2A3) and actin protein expression in a human T lymphocyte leukemia cell line (Jurkat) after a 6-h or 48-h incubation with 50 μM 6-MP or vehicle (V). The immunoblot is representative of three independent experiments.

Cellular glucose influx is a rate-limiting step of glycolysis, and we observed global shutdown of glucose metabolism in cells exposed to 6-MP. Therefore, we asked whether 6-MP can reduce glucose uptake to cause energetic stress and metabolic disruption. Upon measuring the cellular uptake of ^3^H-2-Deoxyglucose (a radioactive glucose analogue phosphorylated by hexokinases but that cannot undergo glycolysis) to monitor glucose influx, we observed no difference between vehicle (V) and 6-MP (Figure 5A), suggesting that 6-MP does not modify glucose uptake. The expression of SLC2A1, also known as Glut1 (glucose transporter 1; the main glucose transporter involved in metabolic reprogramming [36, 37]), was weakly and very transiently increased upon 6-MP exposure (Figure 5B), indicating that 6-MP has no effect on glucose influx; in addition, Glut3 expression was not impacted by 6-MP (Figure 5C). Together, these results indicate that the metabolic alterations associated with 6-MP are likely not caused by inhibition of cellular glucose uptake.

## DISCUSSION

We provide for the first time a comprehensive characterization of the impact of 6-MP exposition in proliferating T cell metabolism. Our results indicate that 6-MP produces an early drop in ATP production followed by transcriptional reprogramming of genes involved in glycolysis, glutaminolysis and nucleotide synthesis; these changes are similar to those observed upon energetic stress. These metabolic alterations are associated with the inhibition of the metabolic checkpoints mTORC1, HIF-1α and Myc are functionally characterized by a reduction of the glycolytic and glutaminolytic fluxes. As a unifying mechanism, we propose that, following early ATP depletion occurring during 6-MP treatment, the metabolic sensor AMPK is activated by phosphorylation, and, in turn, inhibits mTORC1. Besides the inhibition of purine synthesis, the biochemical basis for rapid ATP depletion induced by 6-MP remains to be established. Since mTORC1 regulates the expression of Myc and HIF-1α, mTORC1 inhibition upon ATP depletion may induce the inhibition of Myc and HIF-1α expression. Consequently, a marked reduction of the glycolytic and glutaminolytic fluxes is observed due to a decrease of the expression of enzymes and transporters under the control of Myc and HIF-1α involved in glycolysis such pyruvate kinase M and in glutaminolysis such as GLS1 and 2. In addition, ATP depletion induced by 6-MP could impact glycolytic and glutaminoytic fluxes as a result of the inhibition of ATP-dependent enzymes, such as hexokinase.

These results provide new insights into how incubation with 6-MP, which induces ATP depletion, promotes metabolic stress and adds a possible mechanism by which 6-MP inhibits cell proliferation. In addition, this finding underscores the critical role of ATP production in proliferating T cells, including the Jurkat T cell line [38]. Jurkat cells have high intracellular ATP concentration and generate more extracellular ATP than resting T cells. As a result, increased purinergic signaling through P2X receptors elevates the baseline levels of cytosolic Ca^2+^ in Jurkat cells, which fosters proliferation [38]. Consistent with this, we have recently demonstrated that 6-MP-resistant lymphoblastoid cell lines grow more slowly than 6-MP-sensitive cells, suggesting the importance of basal proliferation rate of cells in anticipating the antiproliferative action of 6-MP [15]. Together, these results underscore the critical role of the maintain of ATP homeostasis in leukemic T cells and offer the opportunity to reassess the beneficial effects of 6-MP as a therapeutic approach in association with other antiproliferative agents that would target multiple metabolic pathways crucial for cell proliferation. In addition, it would be interesting to determine if variations in key molecules regulating glycolysis and glutaminolysis could be surrogate biomarkers of the interindividual variability in the clinical response to 6-MP by and if so monitoring metabolic reprogramming could be useful to predict 6-MP efficacy.

Whether the effect of 6-MP on the metabolic reprogramming of Jurkat T cells can be generalize to other situations during which T cells rapidly grow such as naive T cells upon antigen presentation (that is, in a non-oncological setting) remains to be demonstrated. Our results cannot be directly translated into medical situation other that leukemia because Jurkat cells may not be informative of normal T cell responses. This cell line is not dependent on T-cell receptor (TCR) signals for nutrient utilization, cell cycle progression and growth. In addition, due to phosphatase and tensin homolog (PTEN) loss, Jurkat T cells have a deregulated PI3K/Akt/mTOR pathway activation with constitutively elevated Akt activity. Hence, our findings are relevant for leukemia-related situations, but cannot be directly extrapolated into adaptive immunity. However, when accounting for the role of metabolic checkpoints, including HIF-1α and mTOR, and metabolic reprogramming in naive T-cell polarization and proliferation [39–41], it is reasonable to envision that the immunosuppressive properties of 6-MP, are, at least partially, explained by its effects on ATP production. Along these lines, deciphering the impact of other immunosuppressive agents on metabolic reprogramming, including mycophenolate mofetil which inhibits the rate-limiting enzyme of *de novo* purine synthesis, could yield new insights on its mechanisms of action with important therapeutic implications. Indeed, identifying molecular circuitries that regulate cell metabolism and could eventually be targeted by immunosuppressive drugs is a strong approach to design drugs that block metabolic checkpoints in a complementary manner.

Nevertheless, the impact of 6-MP on metabolic reprogramming appears to be cell-dependent, as opposing effects have been reported. 6-MP induces stabilization of HIF-1α in the HepG2 hepatocarcinoma cell line [21], whereas 6-MP decreases HIF-1α protein expression in our model. Regarding glucose uptake, in the L6 model of rat skeletal muscle, 6-MP increases glucose uptake and Glut4 (SLC2A4, solute carrier family 2, member 4) translocation to the cell surface, a process mediated by NR4A3 [42]. The fact that T cells do not express Glut4 [37] may explain why 6-MP does not promote increased glucose uptake in our model. Since the precise mechanisms that cause the adverse effects of 6-MP (e.g., acute liver and pancreatic injuries, sinusoidal epithelial damage (peliosis)) are mostly unknown, one cannot exclude that metabolic perturbations induced by 6-MP in metabolically sensitive cells could be involved in the occurrence of these side effects. In addition, multiple mechanisms are likely involved in the regulation of critical metabolic pathways by 6-MP, and which may be cell-type dependent. For example, we observed that 6-MP activated autophagy, as a probable result of mTOR inhibition and ATP depletion, whereas in HCT116 colorectal cancer cells and HEC59 human endometrial cancer cells, 6-TG-induced DNA damage and P53 activation drove autophagy [43]. Nevertheless, these distinct mechanisms are not exclusive, and whether P53 signaling is activated upon 6-MP exposure remains to be determined. The impact of the energetic stress induced by 6-MP on cell viability, and in comparison with established mechanisms such as incorporation 6-TG in DNA, Rac-1 inhibition and *de novo* purine synthesis remains to be established. However, the specific delineation of the impact of the energetic stress induced by 6-MP is complicated by the interdependence of the signaling pathways activated in the same time by 6-MP. For example, DNA damage induced by 6-TG activate P53 signaling, which, in turn, can impact metabolic checkpoints and cell metabolism. In addition, the metabolic changes induced by 6-MP correspond to an adaptive process, and AMPK activation, mTOR inhibition and autophagy have prosurvival, rather than proapoptotic properties. Since these “beneficial” pathways are activated in parallel to prodeath pathways (such as DNA damage and P53) the net effect on cell viability will depend on many parameters, including cell type, the intensity and the duration of the stress, and the coexistence of sensitizing factors.

In conclusion, our findings offer new insights on the cellular effects of 6-MP on metabolic alterations by demonstrating that 6-MP promotes an early energetic stress that impacts proliferation and increases apoptosis in proliferating T cells. The most significant result of our study was the inhibition of the metabolic checkpoints mTOR, HIF-1α and Myc by 6-MP which was associated with global inhibition of glycolytic and glutaminolytic fluxes into the TCA cycle. Finally, our findings might provide an original rational approach to better redesign therapeutic combinations of antiproliferative agents with the aim of controlling cell metabolism by targeting different metabolic pathways.

## MATERIALS AND METHODS

### Cell culture and chemicals

Jurkat T leukemia cells (clone E6-1, Lot Number 60628582, received November 2014) were obtained from the American Type Culture Collection (ATCC, Manassas, VA, USA). Jurkat cells are T lymphocytes obtained from a peripheral blood of 14 years-old male with acute T cell leukemia, and they express CD3 and CD25. In the manuscript, we used the terms “T cells” or “Jurkat cells” indistinctively. Cells were maintained at 37°C in RPMI 1640 medium (ref. A10491-01 from Gibco^®^, Thermo Fisher Scientific, Waltham, MA, USA) supplemented with 10% fetal bovine serum (FBS), 50 U/mL of penicillin and 50 μg/mL of streptomycin. Cell cultures were maintained at a density of 5 × 10^5^ cells/mL. This cell line was mycoplasma-free (Mycoalert Mycoplasma Detection Kit, Lonza, Slough, UK). 6-MP, 5-aminoimidazole-4-carboxamide ribonucleotide (AICAR) and mitomycin C were purchased from Sigma-Aldrich (Rocky Hill, NJ, USA).

Energetic stress was produced in culturing cells for 16 h in a medium SILAC RPMI 1640 (ref. A2494201 from Gibco^®^) without glucose and glutamine and not supplemented with FBS but with 50 U/mL of penicillin and 50 μg/mL of streptomycin.

### Viability studies

Jurkat cells were seeded in 96-well plates (5 × 10^5^ cells/mL), and the relative number of living cells per well was determined by using the CellTiter 96^®^ Aqueous One Solution Cell Proliferation Assay with 3-(4,5-dimethylthiazol-2-yl)-5-(3-carboxymethoxyphenyl)-2-(4-sul-fophenyl)-2H-tetrazolium (MTS) (Promega, Madison, WI, USA) following the manufacturer's protocol.

### Cell apoptosis assay

The cell apoptosis assay was performed as described previously [44]. Jurkat cells were seeded in 12-well plates (5 × 10^5^ cells/mL) and incubated with 50 μM 6-MP or vehicle (V) for 24, 48 or 72 h. After the incubations, apoptosis was analyzed by mixing 25 μL of cell suspension with 25 μL of an ethidium bromide (EB, 500 μg/mL) and acridine orange (AO, 150 μg/mL) mixture. Cell morphology was studied using a fluorescence microscope. Between 100 and 200 cells were counted per condition. Live cells were stained green, and apoptotic cells were stained orange with shrunken and fragmented nuclei. Among all cells counted, the percentage of apoptotic cells was calculated.

### RNA extraction and real-time quantitative polymerase chain reaction (RT-qPCR)

RNA extraction and RT-qPCR was performed as previously described [45]. Briefly, total RNA was extracted using an RNeasy Mini Kit^®^ (Qiagen, Valencia, CA, USA) according to the manufacturer's instructions. Transcription expression levels were assessed by using a SYBR Green RT-qPCR kit with an ABI-PRISM 7900 sequence detector system (Applied Biosystems, Foster City, CA, USA). The fold changes for each tested gene were normalized to the housekeeping gene ribosomal protein L13A (RPL13A). The relative expression of each gene was calculated using the 2^−ΔΔCT^ method [46]. Accordingly, the expression level of a given gene in the control samples (vehicle-treated) using the 2^−ΔΔCT^ method was set to 1. Comparison of the expression of genes after exposition was visualized as heat maps. Primers sequences are listed in Supplementary Table 1.

### Protein extraction and Western blot analysis

Immunoblotting was performed as previously described [47]. Total protein lysates were separated by sodium dodecyl sulphate polyacrylamide (SDS-PAGE) gel electrophoresis in denaturing conditions and transferred to a polyvinylidene fluoride (PVDF) membrane (GE Healthcare, Pittsburgh, PA, USA). They were incubated with Anti-Phospho-p70 S6 Kinase (Thr421/Ser424), Anti-c-Myc, Anti-AMPKα, Anti-Phospho-AMPKα (Thr172) (1:1000, Cell Signaling Technology); or Anti-HIF-1α, Anti-Glut1 (1:1000, Novus Biologicals, Littleton, CO, USA); Anti-Glut3 (1:1000, Novus Biologicals, Littleton, CO, USA); Anti-LC3B (1:2000, Cell Signaling Technology) or Anti-β actin (1:10000, Sigma-Aldrich) and were visualized using horseradish peroxidase-conjugated polyclonal secondary antibodies (Dako (Cambridge, UK) or Cell Signaling Technology Inc (Hitchin, UK)) and detected by an ECL reagent^®^ (GE Healthcare). Digital images were analyzed by ImageJ Software (NIH, Bethesda, MD, USA).

### ATP detection assay

A total of 10^3^ cells per well were plated in 96-well plates, and the ATP levels were measured using an ATPlite 2 steps Kit (PerkinElmer, Waltham, MA, USA) according to the manufacturer's conditions. Luminescence was measured at a microplate luminescence counter Enspire Multilabel Reader 2300 (Perkin Elmer). Each condition was measured in triplicate.

### ADP and AMP detection assay

AMP, ADP and AMP/ATP and ADP/ATP ratios were measured using ATP/ADP/AMP Assay Kit (University at Buffalo, Buffalo, NY, USA) according to the manufacturer's protocol. Luminescence was measured as for ATP detection assay. Each condition was measured in triplicate.

### Lactate measurements

Lactate secretion was measured using the Lactate Colorimetric/Fluorometric Assay Kit (BioVision Inc, Milpitas, CA, USA) in Krebs phosphate buffer (at pH 7.6, 6.3 g/L NaCl, 320 mg/L KCl, 140 mg/L CaCl_2_, 148 mg/L KH_2_PO_4_, 267 mg/L MgSO_4_ and 1.91 g/L NaHCO_3_) according to the manufacturer's instructions. The absorption values were plotted against a lactate standard curve to determine the lactate concentrations in the samples.

### Metabolic assays

Glucose and glutamine oxidation flux were determined by the rate of ^14^CO_2_ released from ^14^C-U-glucose and ^14^C-U-glutamine, respectively. Jurkat cells were treated for 48 h with 50 μM 6-MP. Then, 5 × 10^6^ cells were resuspended in 950 μL of Krebs-Ringer phosphate buffer supplemented with either 5 mM ^14^C-U-glucose (11 GBq/mmol, isotopic dilution 1/1000, Perkin Elmer) or 4 mM ^14^C-U-glutanime (9.69 GBq/mmol, isotopic dilution 1/1000, Perkin Elmer). After a 90-min incubation at 37°C, the reaction was stopped by adding 250 μL of 6 N H_2_SO_4_, and CO_2_ was recovered for 1 h in benzethonium hydroxide. The radioactive CO_2_ was counted by using liquid scintillation (Ultima Gold, Perkin Elmer).

### Glucose uptake

Glucose uptake was measured according to the method described by Hardonnière et al. [48] with some modifications. After the incubation of cells with 50 μM 6-MP for 48 h, 5 × 10^6^ cells were washed with PBS and incubated in 5 mL of RPMI 1640 glucose-free medium supplemented with 10% SVF and 2 mM of glutamine at 37°C for 3 h. After this starvation period, cells were washed and incubated with Krebs-Ringer phosphate buffer for an additional 30 min at 37°C followed by incubation with 0.1 mM ^3^H-2-Deoxyglucose (isotopic dilution of 1:4000) for 10 min at 37°C. After the cells were gently washed twice with ice-cold Krebs-Ringer phosphate buffer, the cell pellets were lysed by adding 250 μL of 0.1 N NaOH. Half of the sample content was transferred into scintillation vials, and the radiolabelled glucose incorporated into the cells was measured by using Ultima Gold and reading the samples on a liquid scintillation counter. The protein content for each condition was assayed by using the remaining half of the sample with a Pierce^™^ BCA Protein Assay Kit (Thermo Fisher Scientific).

### Flow cytometry

#### Cell cycle analysis

A total of 1 × 10^6^ cells per condition were washed with PBS, and the pellets were fixed by 70% ethanol and incubated for 30 min in 400 μl of PBS containing 100 μL IPEGAL 1% (NP-40), 50 μL of RNase (1 mg/mL in PBS with 0.5 mM of EDTA), 100 μL of PI (propidium iodide, 50 μg/mL). Cells were incubated for 10 min at 37°C prior to flow cytometry on a FACSCanto II (BD Biosciences, San Jose, CA, USA) and analysis with FlowJo software (TreeStar, Ashland, Oregon, USA).

### Cell proliferation assay

For the CFSE dilution analysis, 5 × 10^5^ cells/mL were resuspended in PBS. A stock solution of CFSE (10 mM, Invitrogen, Carlsbad, CA, USA) was added to a final concentration of 2.5 μM and incubated with the cells at 37°C for 15 min. After the cells were washed with PBS, medium was added, and the cells were incubated for 30 min at 37°C. Approximately 2 × 10^6^ cells were removed, washed with PBS and fixed with 3.7% PFA for 15 min at 4°C. Afterwards, the cells were washed and resuspended with PBS before they were subjected to flow cytometry on a FACSCanto II (BD Biosciences). The data were analysed with FlowJo software (TreeStar).

### Statistical analysis

The results are expressed as the means ± SD. The distribution of variables is represented using box-and-whisker plots. The distributions are represented using histograms. We used the Mann-Whitney *U* test for nonparametric data comparisons between two groups and a *t*-test to compare the parametric data. Statistical analysis were performed using GraphPad Prism software version 5.0 (Graphpad Software Inc, La Jolla, CA, USA), which was also used to produce the graphs. *P* values < 0.05 were considered statistically significant.

## SUPPLEMENTARY FIGURES AND TABLE


