# Supplementary material for: 6-mercaptopurine promotes energetic failure in proliferating T cells
Source: Oncotarget. 2017 May 16;8(26):43048–60. doi: 10.18632/oncotarget.17889 (PMC5522126; doi:10.18632/oncotarget.17889)
Supplement: Supplementary file 1 [file oncotarget-08-43048-s001.pdf]

6-mercaptopurine promotes energetic failure in proliferating T cells

Supplementary Materials

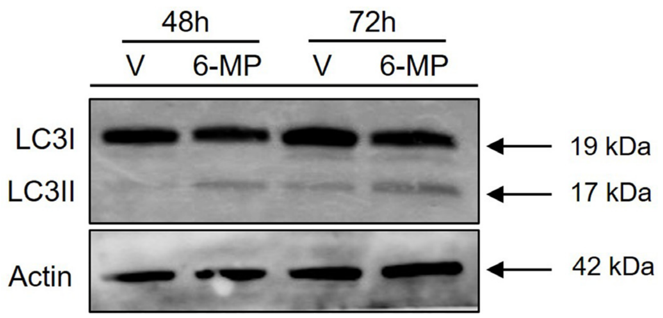

Supplementary Figure 1: LC3 immunoblot upon 6-MP exposure. Immunoblot representing LC3I and LC3II after 48 and 72 h incubation with 50 µM 6-MP or vehicle (V). The immunoblot is representative of three independent experiments.

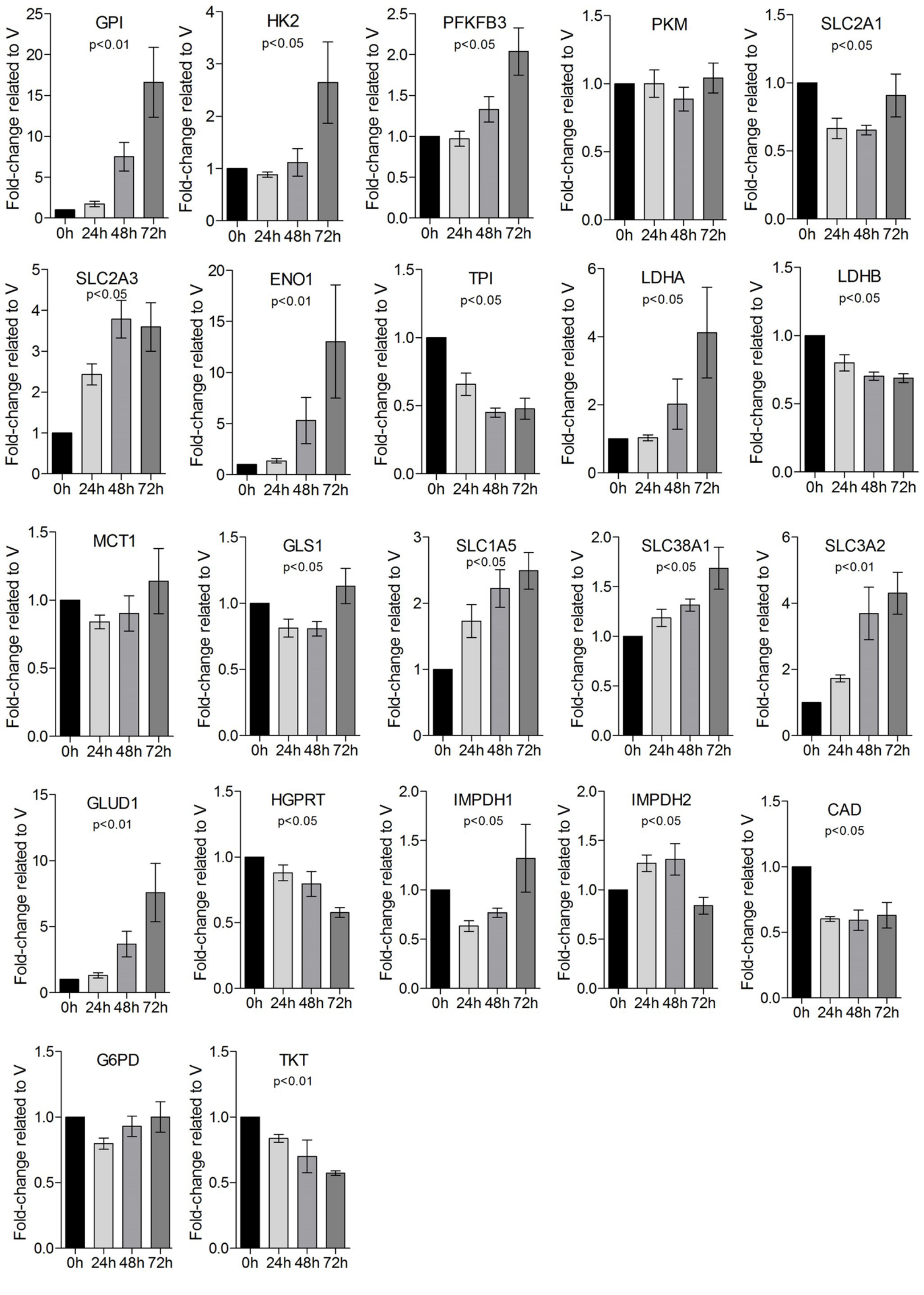

Supplementary Figure 2: Histograms of each gene implicated in glycolysis, glutaminolysis and nucleotide synthesis after 24, 48 and 72 h of incubation with 6-MP. Histograms are representative of four independent experiments. One-way ANOVA statistical analysis was performed.

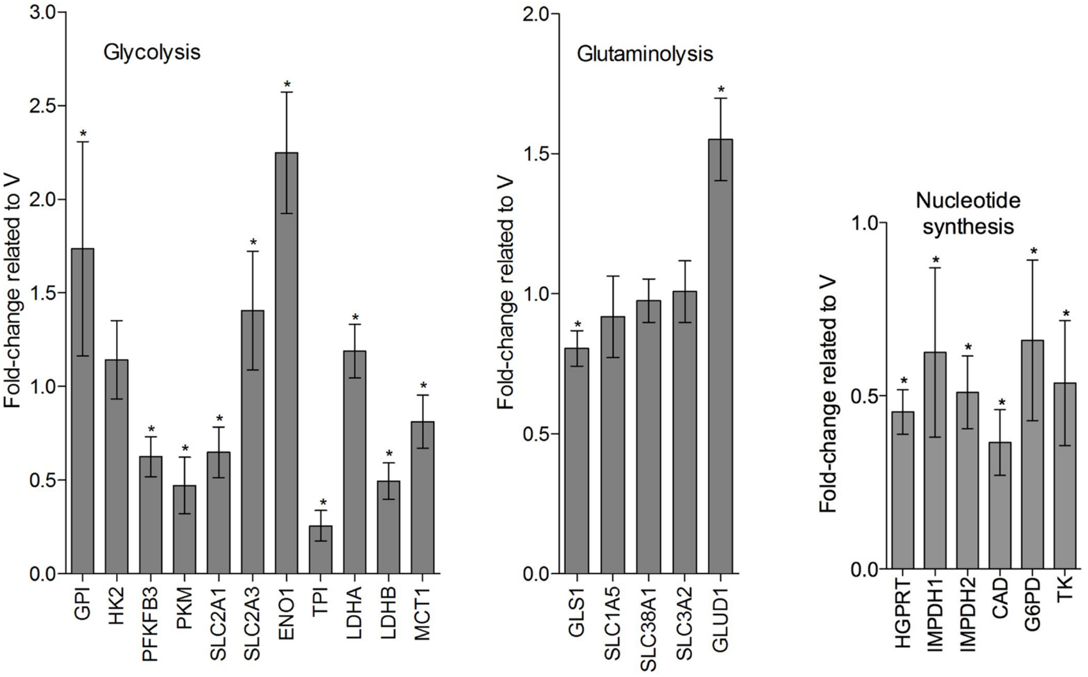

Supplementary Figure 3: Histograms of each gene implicated in glycolysis, glutaminolysis and nucleotide synthesis after 16 h of glucose and glutamine deprivation. Histograms are representative of four independent experiments.

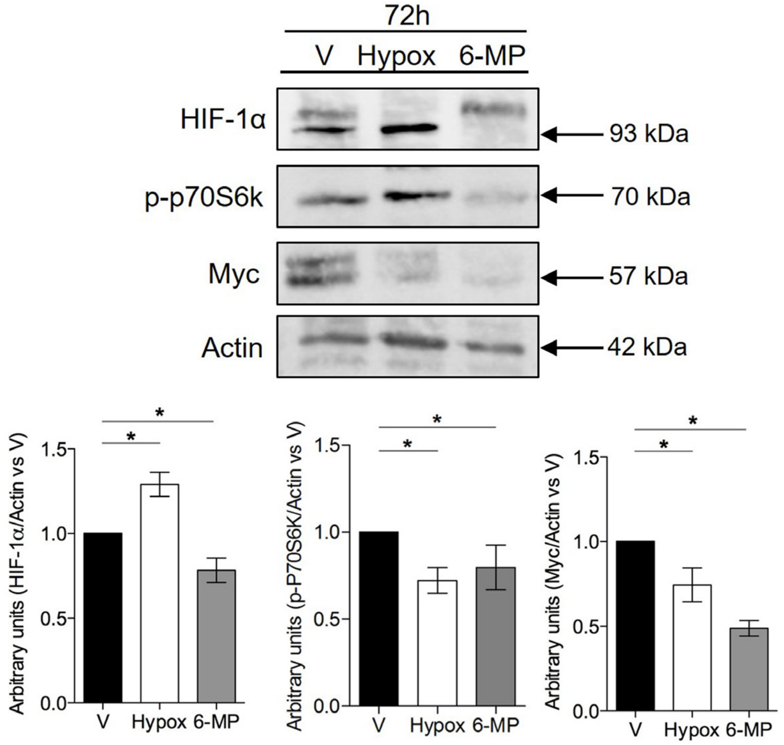

Supplementary Figure 4: Immunoblot after incubation with 6-MP. (Top) Immunoblot representing phospho-p70S6K (70 kDa ribosomal protein S6 kinase 1), HIF-1α (hypoxia inducible factor 1α), Myc, and actin protein expression in a human T lymphocyte leukaemia cell line (Jurkat) after 72 h of incubation 50 µM 6-MP or vehicle (V). A 24-h exposure to hypoxia was used as a control. The immunoblot is representative of four independent experiments. (Bottom) Histograms representing the densitometric analysis of the immunoblots.

Supplementary Table 1: Primer sequences used for RT-PCR analysis

Gene name	Primer sequence
GPI	F 5′-ccaccagcagacacacatca-3′<br>R 5′-cctgtgcactagtcggcttc-3′
HK2	F 5′-tggcagaccttcatcttccttc-3′<br>R 5′-aaacacacagtggaaactggc-3′
PFKFB3	F 5′-ggtgtgcgacgaccctac-3′<br>R 5′-gtacacgatgcggctctg-3′
PKM	F 5′-cgggataaccttgaggctga-3′<br>R 5′-gaagagatccggagccacg-3′
SLC2A1	F 5′-tatgtggagcaactgtgtggt-3′<br>R 5′-tccggcctttagtctcagga-3′
SLC2A3	F 5′-gacacagaaggtcaccccag-3′<br>R 5′-gacgaagagtccgacggaaa-3′
ENO1	F 5′-ttcggctcaccggtcctatc-3′<br>R 5′-ggagagccgtcactcattcc-3′
TPI	F 5′-cgttgggggaaactggaagat-3′<br>R 5′-aaaccacctcggtgtcggc-3′
LDHA	F 5′-acgtcagcatagctgtgcaa-3′<br>R 5′-aggaatcgggaatgcacgtc-3′
LDHB	F 5′-gcctcctctctcctgtgcaa-3′<br>R 5′-cctcttcttccgcaactggt-3′
MCT1	F 5′-ggttataaggcagcctcgc-3′<br>R 5′-ttgcttgttccagtacccacg-3′
GLS1	F 5′-tccccaaggacaggtggaat-3′<br>R 5′-gaggtgtgtggacttggt-3′
SLC1A5	F 5′-tggactggctagtcgaccg-3′<br>R 5′-gggcagctcactcttcactt-3′
SLC38A1	F 5′- gggattttgggactcgcctt-3′<br>R 5′- tacaccatgcagcctgtttct-3′
SLC3A2	F 5′- gggcctggactcttctccta-3′<br>R 5′- ggcccacatccccaaagtta-3′
GLUD1	F 5′- gtaactgcatggcttaacctgg-3′<br>R 5′- tctgggcagctcacaataaag-3′
HGPRT	F 5′- ttgctttccttggtcaggca-3′<br>R 5′- atccaacacttcgtggggtc-3′
IMPDH1	F 5′- gtctgtccttcggtccatga-3′<br>R 5′- gccgcttttcgtaagagtgc-3′
IMPDH2	F 5′-tggaggcaatgtggtcactg-3′<br>R 5′-gccagcacttcctgcgtaa-3′
CAD	F 5′- ggagtttgcagtccttcccg-3′<br>R 5′- gccggtttgaaacaccactt-3′
G6PD	F 5′-cgacgacgacgacgaagcgcaga-3′<br>R 5′-ggccaggtcacccgatgcac-3′
TKT	F 5′-tgtgtccagtgcagtagtgg-3′<br>R 5′-acacttcatacccgccctag-3′
RPL13A	F 5′-cctggaggagaagaggaaagaga-3′<br>R 5′-gaggacctctgtgtatttgtcaa-3′
